# Public health advocacy to reinstate reproductive rights of Particularly Vulnerable Tribal Groups (PTGs) in Chhattisgarh

**DOI:** 10.1186/1753-6561-6-S5-P1

**Published:** 2012-09-28

**Authors:** Sulakshana Nandi, JP Mishra, Kanica Kanungo, Hemnarayan Manikpuri, Chandrakant Yadav, Gangaram Paikra, Vivek Upadhyay

**Affiliations:** 1 Public Health Resource Network, Chhattisgarh, India; 2 State Health Resource Centre, Chhattisgarh, India; 3 Lok Astha Sewa Sansthan, Chhattisgarh, India; 4 Astha Samiti, Chhattisgarh, India; 5 Pahari Korwa Mahapanchyat, Chhattisgarh, India

## Introduction

*Baigas*, *Pahari Korwas*, *Abujhmarias*, *Birhors* and *Kamars* have been designated as Particularly Vulnerable Tribal Groups (PTGs) in Chhattisgarh. These communities face high levels of poverty, malnutrition, and limited access to health and nutrition services, manifested in high mortality rates. As a strategy for curtailing the once decreasing population of PTGs after 1979, Madhya Pradesh/Chhattisgarh state government restricted sterilisation among these tribal groups, attempting an increase in birth rate rather than a decrease in the mortality rate.

Today, PTGs, unable to cope with their poverty and large families, are demanding the right to choose their family size and gain access to family planning services. Public Health Resource Network (PHRN) and State Health Resource Centre, Chhattisgarh (SHRC) along with local organisations are attempting to impact policy on this issue through evidence building, community mobilisation and media advocacy.

## Methods

We surveyed 1200 households of *Baiga*, *Pahari Korwa* and *Kamar* communities in Kawardha, Sarguja and Gariaband districts of Chhattisgarh to understand access of PTGs to health and nutrition services, status of their livelihoods, ownership of resources, and land tenure under Forest Rights Act. We organised meetings (*sammelans*) of PTG communities and PTG Mitanins (community health workers) at the block level to present study findings, case studies and facilitate articulation of demands by the PTG community members in the presence of government officials and media. We also examined the 1979 government order related to sterilisations among PTGs. We organised advocacy on the issues that emerged through the study and the *sammelan* through mass media. Advocacy for policy change was undertaken using the emerging evidence and demands of the community.

## Results

We found that the access of PTG families to various health services is limited. Only 32% of the PTG families interviewed had received *Rashtriya Swasthya Bima Yojana* cards (RSBY – an insurance programme for people below poverty line). Of these, only 4% had used the card though 85% were *Antyodaya* beneficiaries. We also found that in only 6% of the families, women obtained financial incentives under the *Janani Suraksha Yojana* scheme.

We shared the study findings during the *sammelans* and the PTGs demanded access to permanent family planning methods, expressing their inability to sustain large families in their current state of impoverishment. Many women had undergone permanent sterilisation under a false name or caste. The administrators including district Collectors, block officials and health department personnel were sensitised towards the plight of the PTGs and their demands.

The order of 1979 of the Government of Madhya Pradesh related to sterilisations for PTGs was not available at the state level and was finally accessed at the district. Analysis of the order showed that the order itself did not propose a ‘ban’ on permanent sterilisations for PTGs. It had been interpreted and operationalised as a ban, which continues till today.

The findings were shared with media. They took up this issue in a big way and many newspapers published follow-up investigative reports after visiting various PTG communities. Based on this, SHRC Chhattisgarh formally wrote to the director of health services of Chhattisgarh to revoke this order.

## Discussion

PTGs are one of the most impoverished of tribal communities whose traditional livelihoods have been destroyed. Though the issues of health and nutrition are very severe among these groups, they are the ones with least access to health and nutrition services, manifested in high malnutrition and mortality rates. In such a situation, the negative implications of the ‘ban’ on sterilisation, on their lives are enormous.

Denial of access to family planning services and of the right to choose one’s family size is a denial of basic human right and therefore has to be corrected at the earliest. It has to go hand-in-hand with interventions to improve their livelihoods, reduce malnutrition and improve their access to nutrition and health schemes and basic health services. Organisations of PTGs themselves should be empowered to put forward these demands and participate in planning and monitoring initiatives meant for them. This process has started in the blocks where this initiative took place and gradually a consensus is building up within the government in Chhattisgarh on revoking the order and reinstating the reproductive rights of the PTGs.

## Competing interests

Authors declare that they have no conflict of interest.

## Funding statement

The study and the *sammelans* were funded under the Chhattisgarh National Rural Health Mission Programme Implementation Plan 2011-12 under the initiative for vulnerable groups.

